# Estimation of primary radiation output for wide‐beam computed tomography scanner

**DOI:** 10.1002/acm2.12598

**Published:** 2019-05-02

**Authors:** Atsushi Fukuda, Pei‐Jan P. Lin, Nao Ichikawa, Kosuke Matsubara

**Affiliations:** ^1^ Department of Radiology Virginia Commonwealth University Medical Center Richmond VA USA; ^2^ Preparing Section for New Faculty of Medical Science Fukushima Medical University Fukushima Japan; ^3^ Department of Radiology Shiga General Hospital Shiga Japan; ^4^ Department of Quantum Medical Technology, Graduate Course of Medical Science and Technology, Division of Health Science Kanazawa University Graduate School of Medical Sciences Ishikawa Japan; ^5^ Department of Quantum Medical Technology, Faculty of Health Sciences Kanazawa University Ishikawa Japan

**Keywords:** beam width, CT, primary radiation output, scattered radiation

## Abstract

**Purpose:**

To estimate in‐air primary radiation output in a wide‐beam multidetector computed tomography (CT) scanner.

**Materials and methods:**

A 6‐cc ionization chamber was placed free‐in‐air at the isocenter, and two sheets of lead (1‐mm thickness) were placed on the bottom of the gantry cover, forming apertures of 40–80 mm in increments of 8 mm. The air‐kerma rate profiles were measured with and without the apertures (${\dot{K}}_{\text{w}-\text{A}}$, ${\dot{K}}_{\text{w}/\text{o}-\text{A}}$) for 4.8 s at tube potentials of 80, 100, 120, and 135 kVp, tube current of 50 mA, and rotation time of 0.4 s. The nominal beam width was varied from 40 to 160 mm in increments of 40 mm. Upon completion of data acquisition, the ${\dot{K}}_{\text{w}/\text{o}-\text{A}}$ were plotted as a function of the measured beam width, and the extrapolated dose rates (${\dot{K}}_{0-\text{w}/\text{o}-\text{A}}$) at zero beam width were calculated by second‐order least‐squares estimation. Similarly, the ${\dot{K}}_{\text{w}-\text{A}}$ were plotted as a function of the radiation field (measured beam width × aperture size at the isocenter), and the extrapolated dose rates (${\dot{K}}_{0-\text{w}-\text{A}}$) were compared with the ${\dot{K}}_{0-\text{w}/\text{o}-\text{A}}$.

**Results:**

The means and standard errors of the ${\dot{K}}_{\text{w}/\text{o}-\text{A}}$ with 40‐, 80‐, 120‐, and 160‐mm nominal beam widths at 120 kVp were 10.94 ± 0.01, 11.13 ± 0.01, 11.22 ± 0.01, and 11.31 ± 0.01 mGy/s, respectively, and the ${\dot{K}}_{0-\text{w}/\text{o}-\text{A}}$ was reduced to 10.67 ± 0.02 mGy/s. The ${\dot{K}}_{0-\text{w}-\text{A}}$ of 40‐, 80‐, 120‐, and 160‐mm beam widths were reduced to 10.6 ± 0.1, 10.6 ± 0.2, 10.5 ± 0.1, and 10.6 ± 0.1 mGy/s and were not significantly different from the ${\dot{K}}_{0-\text{w}/\text{o}-\text{A}}$.

**Conclusions:**

A method for describing the in‐air primary radiation output in a wide‐beam CT scanner was proposed that provides a means to characterize the scatter‐to‐primary ratio of the CT scanner.

## 1 INTRODUCTION

Measurement of radiation output is an important part of quality assurance (QA) for computed tomography (CT) scanners, and the CT dose index (CTDI) has been utilized for over three decades.^1^ With recent advances in technology, the beam width of some CT scanners has been increased to 160 mm.^2^ The use of conventional CTDI_100_ for 160‐mm wide x‐ray beam is now less valid because (a) the beam width exceeds (is wider than) the length of the pencil chamber, (b) the use of just one CTDI phantom is not long enough in z‐axis to adequately provide scatter tails from CTDI phantom, and (c) the CTDI_vol_ is not conceptually appropriate for stationary cone beam CT or for perfusion studies or CT fluoroscopy.^3^ Additionally, the higher gantry rotation speed also decreases the accuracy of CTDI_100_ measurement.^4^.

One of the solutions for the evaluation of radiation output in a wide‐beam CT scanner is to use a farmer‐type ionization chamber and measure the air‐kerma rate free‐in‐air (${\dot{K}}_{\text{air}}$) at the isocenter without CTDI phantom.^2^, ^5^ Because these measurements are carried out with little scattered radiation, they are utilized not only for the determination of ${\dot{K}}_{\text{air}}$, but also for characterization of the half‐value layer (HVL)^6^, ^7^ and the bow tie filter profile.^8^ However, since ${\dot{K}}_{\text{air}}$ increases as a function of the beam width while the tube potential and tube current remain constant, it must still include the scattered radiation.^9^

Although the reduction in scattered radiation is an important factor for measurement of ${\dot{K}}_{\text{air}}$, to the best of our knowledge, there have been no published studies extracting the scattered radiation from the measured ${\dot{K}}_{\text{air}}$. The ionization chamber placed at the isocenter simultaneously detects the primary and the scattered radiation. The scattered radiation increases as a function of the beam width, while the primary radiation remains constant. Conversely, it can be hypothesized that the contribution of scattered radiation could be removed through extrapolation of ${\dot{K}}_{\text{air}}$ at the zero beam width (${\dot{K}}_{0}$).^10^ Then, the ${\dot{K}}_{0}$ at any beam width would be independent of the beam width and would represent the primary radiation output of the CT system.

## 2 METHODS

### 2.1 Measurement of x‐ray beam width

The x‐ray beam width is necessary to extrapolate ${\dot{K}}_{0}$ in this study. It can be determined with conventional film, Gafchromic film, or computed radiography (CR) photostimulable phosphor plate.^11^ Previous work has shown that the CR system is promising for application to beam width measurement because of its accuracy and widespread availability in diagnostic radiology.^12^

#### 2.1.1 Relationship between CR pixel value and *K*_air_

An Aquilion ONE ViSION Edition CT scanner (Canon Medical Systems, Nasu, Japan) and an FCR PROTECT CS CR system (Fujifilm Co., Ltd., Tokyo, Japan) were employed for these experiments. A CR cassette (24 cm × 30 cm) in conjunction with a photostimulable phosphor plate (Imaging Plate Cassette Type CC and IP ST‐VI; Fujifilm Co., Ltd., Tokyo, Japan) was also utilized in this study. Because CT scanning conventionally provides a higher radiation dose to the photostimulable phosphor plate, an appropriate exposure technique is required to avoid saturation of the CR pixel value.^13^ The relation between the CR pixel value and the radiation dose was then investigated.

The CR plate was placed at the isocenter on the patient table with the lead side down. The radiation exposure was taken under axial scanning (tube potential 80 kVp, tube current between 10 and 40 mA at 5‐mA intervals, rotation time 0.275 s, nominal beam width 80 mm, small focus, and medium bow tie filter). Upon completion of the radiation exposures, the CR plate was processed with a fixed mode with a latitude of 4 and sensitivity of 5 using the AVE4.0 test menu in the CR system to avoid any manipulation of raw data.^12^ The CR pixel values at the beam center were measured using ImageJ (National Institute of Mental Health, Bethesda, MD, USA) and plotted as a function of the tube current.

After the relation between the CR pixel value and the tube current was verified for the CR system, the relation between *K*_air_ and tube current was investigated. A 6‐cc ionization chamber (10X6‐6, Radcal, Monrovia, CA, USA) calibrated for RQR‐5 beam quality was suspended free‐in‐air at the isocenter of the CT system, and *K*_air_ was measured under the exact same scanning protocol. Upon completion of these data acquisitions, *K*_air_ was superimposed on the graph of the CR pixel value at the beam center as a function of the tube current. After applying a logarithmic transformation of *K*_air_, the coefficient of determination (R^2^) for log‐linear relationship between CR pixel value and *K*_air_ was calculated below the saturation level.

#### 2.1.2 Measurement of radiation profile

After linearity between the CR pixel value and *K*_air_ had been verified for the CR system, the nominal beam widths of 40, 80, 120, and 160 mm were evaluated. The CR plate was placed at the isocenter on the patient table with the lead side down, and the exposures were taken under axial scanning (tube potential 80 kVp, tube current 10 and 20 mA, rotation time 0.275 s, and bow tie filter medium). The two‐exposure technique was utilized for the determination of full width at half maximum (FWHM), which represents the beam width.^14^ The first exposure (20 mA) was taken for determination of the maximum CR pixel value at the beam center. The second exposure (10 mA) was one‐half of the first exposure and was performed for determination of the half‐maximum exposure level in the first profile. Finally, the FWHM was measured as the distance of the half‐maximum CR values in the first profile.

### 2.2 Estimation of beam width independent in‐air radiation output

**Figure 1:**
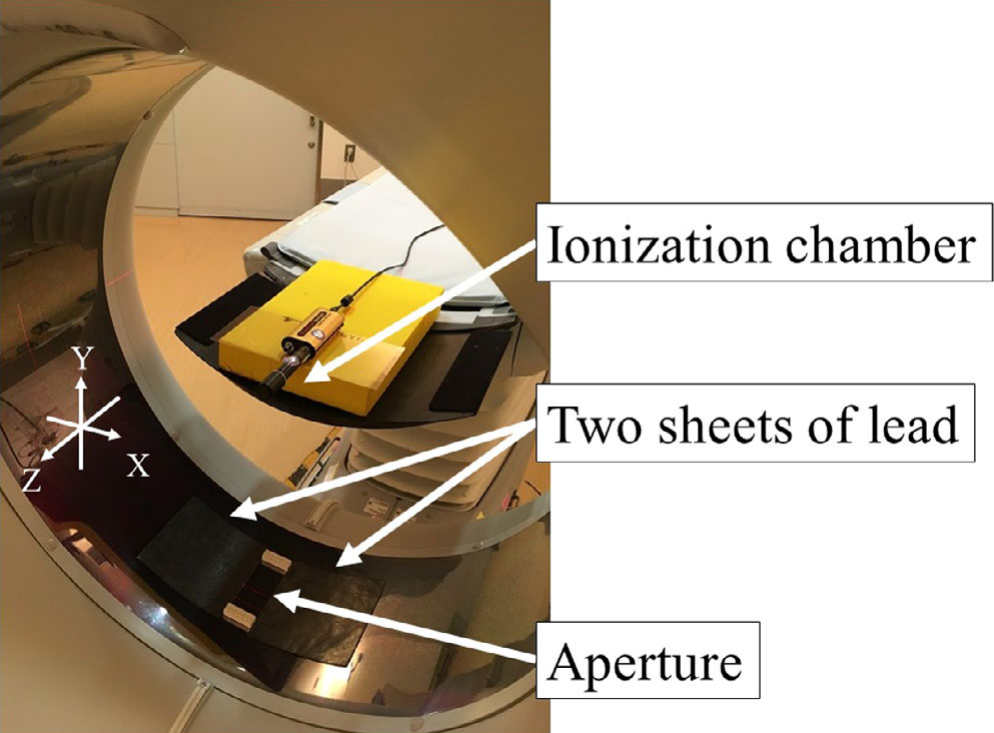
Experimental arrangement for measurement of the ${\dot{K}}_{\text{w}/\text{o}-\text{A}}$ and ${\dot{K}}_{\text{w}-\text{A}}$. A 6‐cc ionization chamber was suspended free‐in‐air at the isocenter of the CT system, and two sheets of lead (160 mm × 160 mm × 1 mm) were placed on the bottom of the gantry cover to form apertures (40, 48, 56, 64, 72, and 80 mm). The apertures were set to collimate the radiation beam along the x‐axis.

A 6‐cc ionization chamber (10X6‐6, Radcal, Monrovia, CA, USA) calibrated for RQR‐9 beam quality was employed in this study. The sensitive volume is 38 mm × 25 mmφ, and the sampling rate is 10 kHz. It was suspended free‐in‐air at the isocenter of the CT system (see Fig. 1), and two sheets of lead (160 mm × 160 mm × 1 mm) were placed perpendicularly to the scan beam width on the bottom of the gantry cover. The XYZ coordinate system was used to express the position relative to the scanner, the horizontal x‐axis, and the vertical y‐axis. The rotation axis was coincident with the z‐axis. The lead sheets were used to create an aperture between 40 and 80 mm (in increments of 8 mm) in the x‐direction. The lead sheets can be seen in Fig. 1. The beam width along the z‐axis was preset at the control console. The ionization chamber was connected to a laptop computer via the Accu‐Gold electrometer (Radcal, Monrovia, CA, USA). Accu‐Gold2 software captured the signal and displayed the radiation waveform for analysis. The ${\dot{K}}_{\text{air}}$ profiles were measured under CINE mode (without table translation) for 4.8 s at tube potentials 80, 100, 120, and 135 kVp, tube current 50 mA, rotation time 0.4 s, and bow tie filter medium. The nominal beam width was varied from 40 to 160 mm in increments of 40 mm. Then, the displayed CTDI_vol_ (IEC60601‐2‐44 ed3.1) was recorded.^15^ Because the Accu‐Gold electrometer with a 6‐cc ionization chamber works as a real‐time dosimeter, the profiles can be divided into two phases, with (${\dot{K}}_{\text{w}-\text{A}}$) and without (${\dot{K}}_{\text{w}/\text{o}-\text{A}}$) the apertures. The ${\dot{K}}_{\text{w}-\text{A}}$ is measured while the x‐ray tube passes at the bottom of the gantry where the aperture is located. The ${\dot{K}}_{\text{w}/\text{o}-\text{A}}$ is the dose rate while the x‐ray tube passes between 7 and 5 o'clock. The ${\dot{K}}_{\text{w}/\text{o}-\text{A}}$ and ${\dot{K}}_{\text{w}-\text{A}}$ were determined using the cursor and magnification tool in Accu‐Gold2 software.

Upon completion of data acquisition, the ${\dot{K}}_{\text{w}/\text{o}-\text{A}}$ were plotted as a function of the measured beam width, and second‐order least‐squares estimation was applied to calculate the extrapolated dose rates (${\dot{K}}_{0-\text{w}/\text{o}-\text{A}}$) at zero beam width. We also calculated the scatter‐to‐primary ratio (SPR) given as a percentage (%), which is defined as

(1) $$SPR=\frac{K_{w/o-A}\;-\;K_{0-w/o-A}}{K_{0-w/o-A}}\times 100$$

Similarly, the ${\dot{K}}_{\text{w}-\text{A}}$ were also plotted as a function of the radiation field at the isocenter, which was calculated as measured beam width × aperture size at the isocenter. Then, the aperture size at the isocenter was calculated from the geometrical data (focus isocenter distance of 600 mm 2 and bore diameter of 780 mm). Finally, the extrapolated dose rates (${\dot{K}}_{0-\text{w}-\text{A}}$) were compared with the ${\dot{K}}_{0-\text{w}/\text{o}-\text{A}}$.

### 2.3 Statistical analysis

Tukey's multiple comparison test was used to evaluate the statistical differences among the ${\dot{K}}_{0-\text{w}/\text{o}-\text{A}}$ and the ${\dot{K}}_{0-\text{w}-\text{A}}$ measured in the CT system. *P*‐values of 0.05 or less were considered to indicate statistically significant differences. All statistical analyses were carried out using the R software package for Windows version 3.5.0 (R Core Team (2018). R: A language and environment for statistical computing. R Foundation for Statistical Computing, Vienna, Austria).^16^

## 3 RESULTS

### 3.1 Measurement of x‐ray beam width

**Figure 2:**
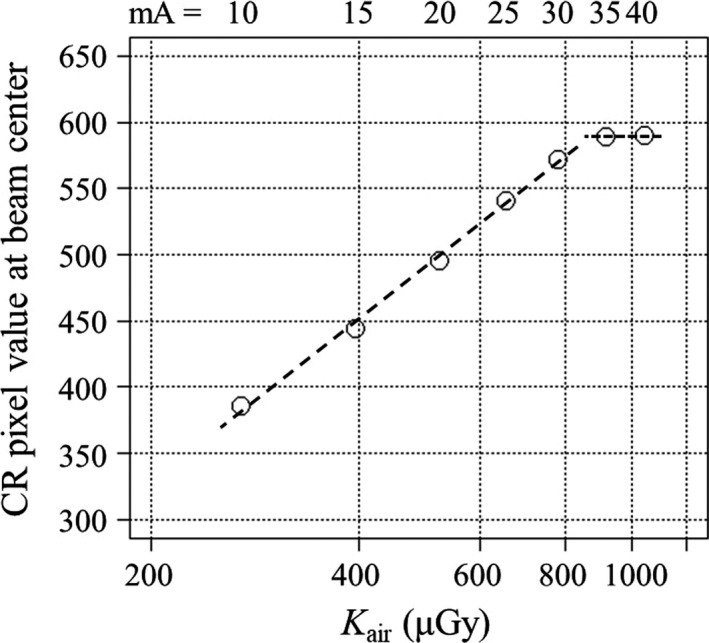
The CR pixel value as a function of *K*_air_ or tube current. CR cassettes (24 cm × 30 cm) in conjunction with a photostimulable phosphor plate were exposed under axial scanning at 80 kVp and rotation time of 0.275 s and were processed with a fixed mode with a latitude of 4 and sensitivity of 5 using the AVE4.0 test menu in the CR system that can avoid any raw data manipulation. It shows a log‐linear relationship between the CR pixel value and *K*_air_ for CT exposure parameters less than or equal to 30 mA (8.25 mAs).

Figure 2 shows the CR pixel values as a function of *K*_air_ or tube current. It shows a log‐linear relationship between the CR pixel value and *K*_air_ for CT exposure parameters less than or equal to 30 mA (8.25 mAs) at 80 kVp, with log‐linear R^2^ of 0.997. Therefore, the two‐exposure technique (10 and 20 mA) is valid for determination of the FWHM.

**Figure 3:**
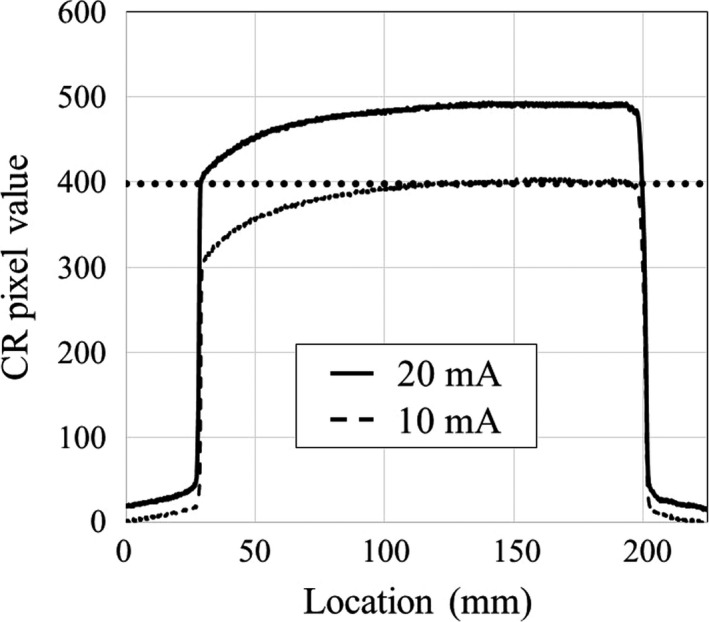
Measurement of 160‐mm beam width by the double‐exposure technique. The half‐maximum CR pixel value in the first exposure (20 mA) is the maximum CR pixel value in the second exposure (10 mA). FWHM was determined as the distance between the half‐maximum CR pixel values in the first exposure (20 mA). FWHM, full width at half maximum.

Figure 3 shows an example of 160‐mm beam width measurement. The half‐maximum CR pixel value in the first exposure (20 mA) is the maximum CR pixel value in the second exposure (10 mA). The FWHMs determined with the double‐exposure method at the 40‐, 80‐, 120‐, and 160‐mm nominal beam widths were 45.6, 85.5, 127.8, and 170.7 mm, respectively.

### 3.2 Estimation of beam width independent in‐air radiation output

**Figure 4:**
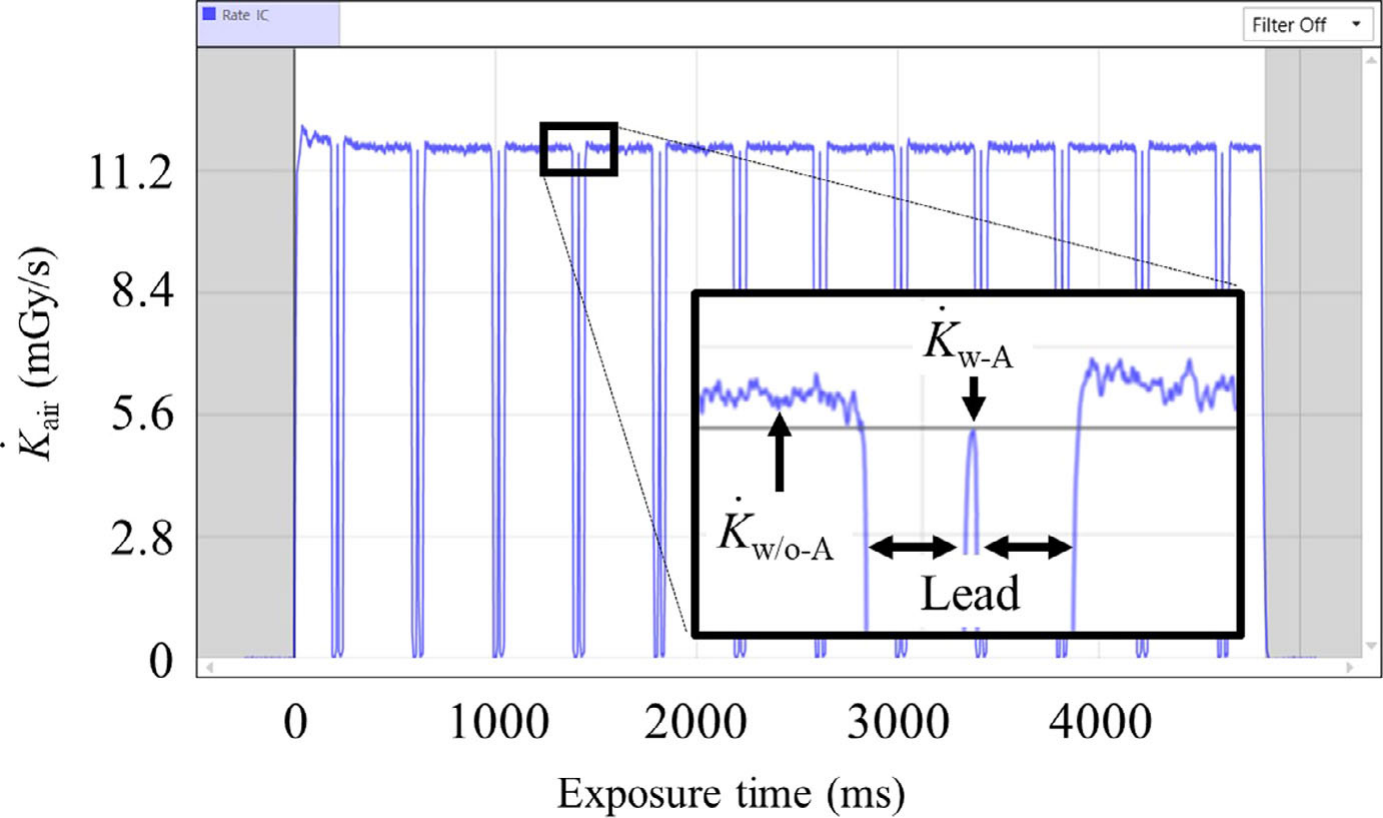
${\dot{K}}_{\text{air}}$ profile for 40‐mm aperture (x‐axis) and 160‐mm beam width (z‐axis) at 120 kVp. The inset shows a magnified view of the rectangle on the waveform showing the ${\dot{K}}_{\text{w}/\text{o}-\text{A}}$ and ${\dot{K}}_{\text{w}-\text{A}}$. The ${\dot{K}}_{\text{w}/\text{o}-\text{A}}$ is the dose rate while the x‐ray tube passes between 7 and 5 o'clock. The ${\dot{K}}_{\text{w}-\text{A}}$ is measured while the x‐ray tube passes by the aperture, which is located on the bottom of the gantry. ${\dot{K}}_{\text{w}/\text{o}-\text{A}}$, air‐kerma rate without aperture; ${\dot{K}}_{\text{w}-\text{A}}$, air‐kerma rate with aperture.

Figure 4 shows the dose rate profile at 120 kVp with 40‐mm aperture (x‐axis) and 160‐mm beam width (z‐axis). A rectangular area is magnified to show the ${\dot{K}}_{\text{w}/\text{o}-\text{A}}$ and ${\dot{K}}_{\text{w}-\text{A}}$, respectively (see the inset). There are 12 ${\dot{K}}_{\text{w}-\text{A}}$ peaks for 4.8 s, and the data in the fourth to eighth rotations were employed to calculate the means and the standard errors of the ${\dot{K}}_{\text{w}/\text{o}-\text{A}}$ and ${\dot{K}}_{\text{w}-\text{A}}$.

**Figure 5:**
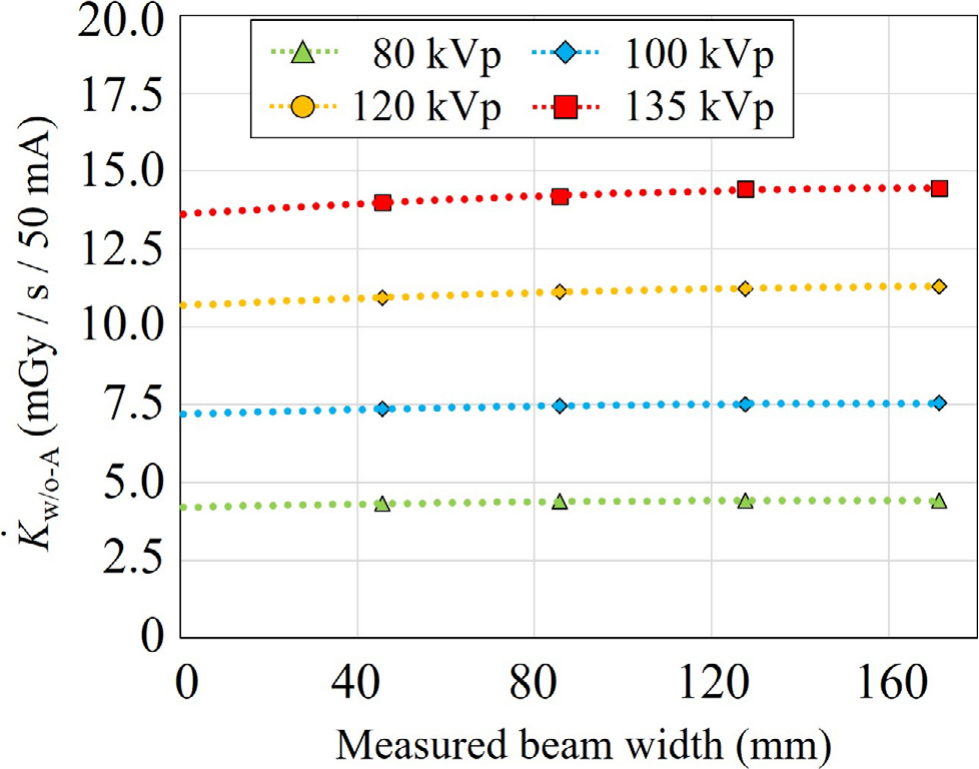
${\dot{K}}_{\text{w}/\text{o}-\text{A}}$ at 80, 100, 120, and 135 kVp as a function of measured beam width. These ${\dot{K}}_{\text{w}/\text{o}-\text{A}}$ were reduced with decrease in the beam width. ${\dot{K}}_{\text{w}/\text{o}-\text{A}}$, air‐kerma rate without aperture.

Figure 5 shows the ${\dot{K}}_{\text{w}/\text{o}-\text{A}}$ at 80, 100, 120, and 135 kVp as a function of the measured beam width. The means and standard errors of the ${\dot{K}}_{\text{w}/\text{o}-\text{A}}$ with 40‐, 80‐, 120‐, and 160‐mm nominal beam widths at 120 kVp were 10.94 ± 0.01, 11.13 ± 0.01, 11.22 ± 0.01, and 11.31 ± 0.01 mGy/s, respectively, and the ${\dot{K}}_{0-\text{w}/\text{o}-\text{A}}$ was reduced to 10.67 ± 0.02 mGy/s. The CTDI_vol_, ${\dot{K}}_{\text{w}/\text{o}-\text{A}}$, ${\dot{K}}_{0-\text{w}/\text{o}-\text{A}}$, and SPR at 80, 100, 120, and 135 kVp as a function of the nominal and measured beam widths are summarized in Table 1. All ${\dot{K}}_{\text{w}/\text{o}-\text{A}}$, as well as the SPR, were reduced with decreasing beam width.

**Table 1:** CTDI_vol_, ${\dot{K}}_{\text{w}/\text{o}-\text{A}}$, ${\dot{K}}_{0-\text{w}/\text{o}-\text{A}}$, and SPR at 80, 100, 120, and 135 kVp as a function of nominal and measured beam widths.

Tube potential (kVp)	Nominal/measured beam width (mm)	CTDI_vol_head/body (mGy/rotation)	${\dot{K}}_{\text{w}/\text{o}-\text{A}}$ (mGy/s/50 mA)	${\dot{K}}_{0-\text{w}/\text{o}-\text{A}}$ (mGy/s/50 mA)	SPR (%)
80	40/45.6	1.3/0.6	4.32 ± 0.01	4.23 ± 0.02	2.2 ± 0.5
	80/85.5	1.2/0.5	4.39 ± 0.01		3.7 ± 0.5
	120/127.8	1.2/0.5	4.41 ± 0.01		4.3 ± 0.4
	160/170.7	1.3/0.6	4.41 ± 0.01		4.2 ± 0.5
100	40/45.6	2.5/1.1	7.35 ± 0.01	7.20 ± 0.02	2.2 ± 0.3
	80/85.5	2.2/1.0	7.47 ± 0.01		3.7 ± 0.4
	120/127.8	2.3/1.1	7.51 ± 0.02		4.2 ± 0.5
	160/170.7	2.4/1.2	7.54 ± 0.02		4.7 ± 0.5
120	40/45.6	3.9/1.8	10.94 ± 0.01	10.67 ± 0.02	2.3 ± 0.2
	80/85.5	3.5/1.6	11.13 ± 0.01		4.1 ± 0.3
	120/127.8	3.5/1.7	11.22 ± 0.01		4.8 ± 0.3
	160/170.7	3.8/2.0	11.31 ± 0.01		5.5 ± 0.2
135	40/45.6	5.1/2.3	14.00 ± 0.01	13.60 ± 0.03	2.7 ± 0.2
	80/85.5	4.6/2.1	14.18 ± 0.01		4.0 ± 0.2
	120/127.8	4.7/2.3	14.43 ± 0.01		5.6 ± 0.3
	160/170.7	5.0/2.6	14.46 ± 0.01		5.8 ± 0.2

Data are given as means and standard errors. Note that CTDI_vol_is displayed under scanning at tube current of 50 mA and rotation time of 0.4 s.

CTDI_vol,_ volume computed tomography dose index; ${\dot{K}}_{\text{w}/\text{o}-\text{A}}$, air‐kerma rate without aperture; ${\dot{K}}_{0-\text{w}/\text{o}-\text{A}}$, air‐kerma rate without aperture extrapolated at the zero beam width; SPR, scatter‐primary ratio.

**Figure 6:**
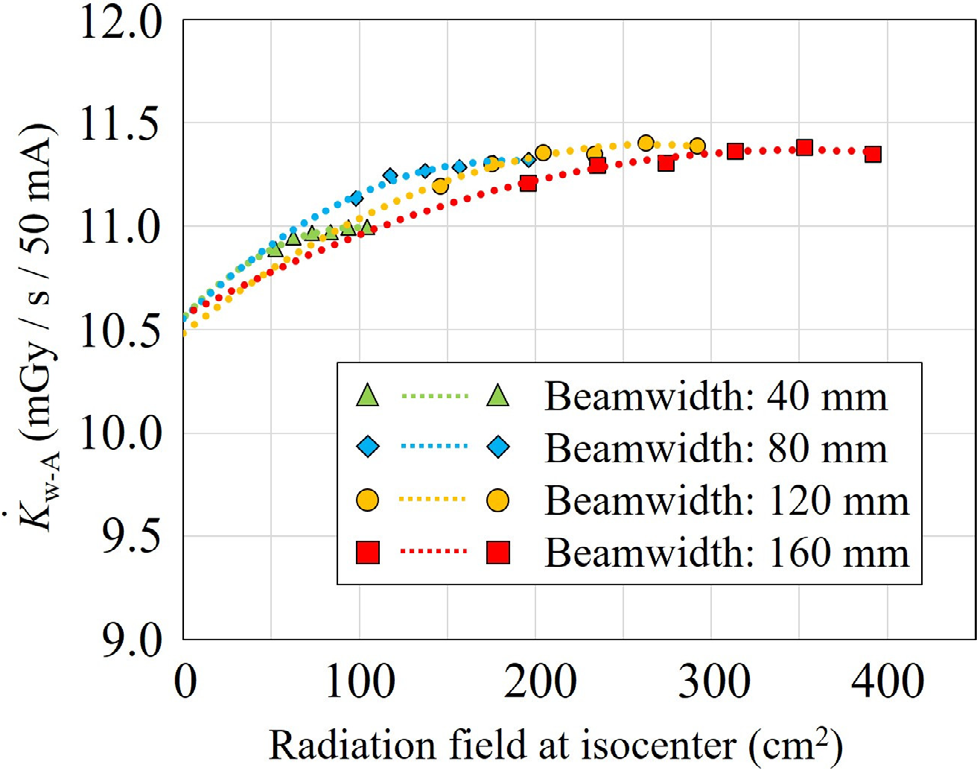
${\dot{K}}_{\text{w}-\text{A}}$ for 40‐, 80‐, 120‐, and 160‐nominal beam widths at 120 kVp as a function of the radiation field at the isocenter. These ${\dot{K}}_{\text{w}-\text{A}}$ were reduced with a decrease in the radiation field, and almost the same ${\dot{K}}_{0-\text{w}-\text{A}}$ were also extrapolated. These values did not significantly differ between beam widths and were comparable to the ${\dot{K}}_{0-\text{w}/\text{o}-\text{A}}$ of 10.67 ± 0.02 mGy/s (no significant differences). ${\dot{K}}_{\text{w}-\text{A}}$, air‐kerma rate with aperture; ${\dot{K}}_{0-\text{w}-\text{A}}$, air‐kerma rate with aperture extrapolated at the zero radiation field at the isocenter; ${\dot{K}}_{0-\text{w}/\text{o}-\text{A}}$, air‐kerma rate without aperture extrapolated at the zero beam width.

Figure 6 and Table 2 show the ${\dot{K}}_{\text{w}-\text{A}}$ of 40‐, 80‐, 120‐, and 160‐mm nominal beam widths at 120 kVp as a function of the radiation field at the isocenter. These ${\dot{K}}_{\text{w}-\text{A}}$ were also reduced with decrease in the radiation field, and the ${\dot{K}}_{0-\text{w}-\text{A}}$ were extrapolated as 10.6 ± 0.1, 10.6 ± 0.2, 10.5 ± 0.1, and 10.6 ± 0.1 mGy/s, respectively. These rates did not significantly differ between beam widths and were comparable to the ${\dot{K}}_{0-\text{w}/\text{o}-\text{A}}$ of 10.67 ± 0.02 mGy/s (no significant differences). However, it is worth mentioning that the standard errors of the ${\dot{K}}_{0-\text{w}-\text{A}}$ are ten times larger than those of the ${\dot{K}}_{0-\text{w}/\text{o}-\text{A}}$, because the ionization chamber is exposed during a much shorter time during the ${\dot{K}}_{\text{w}-\text{A}}$ part of the acquisition compared to ${\dot{K}}_{\text{w}/\text{o}-\text{A}}$. The measurement of the ${\dot{K}}_{0-\text{w}-\text{A}}$ is also tedious and time‐consuming compared with measurement of the ${\dot{K}}_{0-\text{w}/\text{o}-\text{A}}$.

**Table 2:** ${\dot{K}}_{\text{w}-\text{A}}$ as a function of aperture size and ${\dot{K}}_{0-\text{w}-\text{A}}$ at 80, 100, 120, and 135 kVp in combination with 40‐, 80‐, 120‐, and 160‐mm nominal beam widths.

Tube potential (kVp)	Nominal/measured beam width (mm)	${\dot{K}}_{\text{w}-\text{A}}$ (mGy/s/50 mA)						${\dot{K}}_{0-\text{w}-\text{A}}$ (mGy/s/50 mA)	Statistically significant difference	
		Aperture size (mm)								
		40	48	56	64	72	80		Against${\dot{K}}_{0-\text{w}/\text{o}-\text{A}}$	Against ${\dot{K}}_{0-\text{w}-\text{A}}$
80	40/45.6	4.25 ± 0.01	4.27 ± 0.01	4.30 ± 0.01	4.31 ± 0.01	4.31 ± 0.01	4.33 ± 0.01	4.1 ± 0.1	N.S.	N.S
	80/85.5	4.44 ± 0.01	4.47 ± 0.01	4.48 ± 0.01	4.47 ± 0.01	4.47 ± 0.01	4.46 ± 0.01	4.2 ± 0.1	N.S.	
	120/127.8	4.46 ± 0.01	4.50 ± 0.01	4.51 ± 0.02	4.54 ± 0.01	4.51 ± 0.01	4.53 ± 0.01	4.1 ± 0.1	N.S.	
	160/170.7	4.41 ± 0.01	4.45 ± 0.01	4.46 ± 0.01	4.48 ± 0.01	4.49 ± 0.01	4.50 ± 0.01	4.2 ± 0.1	N.S.	
100	40/45.6	7.33 ± 0.01	7.36 ± 0.01	7.37 ± 0.02	7.38 ± 0.01	7.41 ± 0.01	7.38 ± 0.01	7.1 ± 0.1	N.S.	N.S.
	80/85.5	7.47 ± 0.02	7.49 ± 0.01	7.59 ± 0.01	7.61 ± 0.01	7.60 ± 0.02	7.63 ± 0.01	6.9 ± 0.1	N.S.	
	120/127.8	7.50 ± 0.01	7.52 ± 0.01	7.55 ± 0.01	7.64 ± 0.01	7.65 ± 0.01	7.67 ± 0.01	7.2 ± 0.1	N.S.	
	160/170.7	7.48 ± 0.01	7.54 ± 0.01	7.61 ± 0.01	7.63 ± 0.02	7.68 ± 0.01	7.72 ± 0.01	7.0 ± 0.1	N.S.	
120	40/45.6	10.89 ± 0.03	10.95 ± 0.02	10.97 ± 0.01	10.97 ± 0.01	10.99 ± 0.02	11.00 ± 0.01	10.6 ± 0.1	N.S.	N.S.
	80/85.5	11.13 ± 0.01	11.24 ± 0.01	11.27 ± 0.02	11.28 ± 0.01	11.31 ± 0.01	11.32 ± 0.01	10.6 ± 0.2	N.S.	
	120/127.8	11.20 ± 0.01	11.30 ± 0.02	11.36 ± 0.01	11.35 ± 0.01	11.41 ± 0.01	11.39 ± 0.01	10.5 ± 0.1	N.S.	
	160/170.7	11.21 ± 0.02	11.30 ± 0.02	11.31 ± 0.02	11.37 ± 0.01	11.38 ± 0.01	11.35 ± 0.01	10.6 ± 0.1	N.S.	
135	40/45.6	14.06 ± 0.01	14.12 ± 0.02	14.14 ± 0.02	14.18 ± 0.03	14.12 ± 0.01	14.17 ± 0.01	13.7 ± 0.2	N.S.	N.S.

	80/85.5	14.18 ± 0.02	14.28 ± 0.02	14.30 ± 0.01	14.29 ± 0.01	14.33 ± 0.02	14.32 ± 0.02	13.7 ± 0.1	N.S.	
	120/127.8	14.26 ± 0.02	14.34 ± 0.01	14.41 ± 0.02	14.46 ± 0.01	14.53 ± 0.02	14.54 ± 0.02	13.6 ± 0.1	N.S.	
	160/170.7	14.34 ± 0.02	14.46 ± 0.03	14.52 ± 0.03	14.56 ± 0.02	14.60 ± 0.01	14.63 ± 0.01	13.6 ± 0.1	N.S.	

Data are given as means and standard errors. Statistically significant differences were computed by Tukey's multiple comparison test among ${\dot{K}}_{0-\text{w}/\text{o}-\text{A}}$ and *K*_0‐w‐A_.

${\dot{K}}_{\text{w}-\text{A}}$, air‐kerma rate with aperture; ${\dot{K}}_{0-\text{w}-\text{A}}$, air‐kerma rate with aperture extrapolated at the zero radiation field at the isocenter; ${\dot{K}}_{0-\text{w}/\text{o}-\text{A}}$, air‐kerma rate without aperture extrapolated at the zero beam width; N.S, not significant.

## 4 DISCUSSION

We measured the ${\dot{K}}_{0-\text{w}-\text{A}}$ and ${\dot{K}}_{0-\text{w}/\text{o}-\text{A}}$ to verify that the *K*_0_ at any beam width is independent of the beam width in a wide‐beam CT scanner. As shown in Tables 1 and 2, the ${\dot{K}}_{0-\text{w}/\text{o}-\text{A}}$ and ${\dot{K}}_{0-\text{w}-\text{A}}$ at 40‐, 80‐, 120‐, and 160‐mm beam widths were not significantly different. From the above examinations, our new technique showed that (a) *K*_0_ indicates the primary radiation output of the CT system, which is independent of the beam width; (b) it can reduce scatter contamination, which affects the accuracy of ${\dot{K}}_{\text{air}}$ measurement; and (c) the ${\dot{K}}_{0-\text{w}/\text{o}-\text{A}}$ is straightforward compared with the ${\dot{K}}_{0-\text{w}-\text{A}}$. Additionally, because the unit of the ${\dot{K}}_{0-\text{w}/\text{o}-\text{A}}$ and ${\dot{K}}_{0-\text{w}-\text{A}}$ is mGy/s, *K*_0_ is independent of the gantry rotation time, which affects the accuracy of CTDI_100_.^4^

Beam width measurement is necessary to extrapolate the ${\dot{K}}_{0-\text{w}/\text{o}-\text{A}}$ and ${\dot{K}}_{0-\text{w}-\text{A}}$, and we employed the double‐exposure technique, the accuracy of which is within the CR system pixel spacing (0.1 mm).^14^ The CR system is accurate for determination of the beam width; however, performing the measurement is tedious and time‐consuming. Some authors have reported radiation dose profiles measured by a small‐cavity ionization chamber or a liquid ionization chamber.^2^, ^9^ Unlike image analysis, such as the CR system, these chambers allow simultaneous measurement of both ${\dot{K}}_{\text{air}}$ and the radiation dose profile to obtain the beam width.

The SPR increased as a function of tube potential and beam width, as shown in Table 1. One reason for the increase may be the probability of Compton scattering in the energy range of diagnostic radiology. The scattered radiation generated in the x‐ray tube assembly increases with opening of the collimator. In addition, backscattered radiation from the imaging detector assembly contributes as the beam width is increased. We found that up to 5.8% of the scattered radiation is included in the measurement of ${\dot{K}}_{\text{air}}$ for the wide‐beam CT scanner. These results are considered to have a minor impact on the estimation of ${\dot{K}}_{\text{air}}$ with 80 mm beam width or less. However, because the measurement uncertainties are increased as a function of the beam width and tube potential, medical physicists should take the SPR into account for the accurate ${\dot{K}}_{\text{air}}$ measurement with 160 mm beam width at tube potentials of 120 and 135 kVp. Because the photon energy of the scattered radiation is lower than that of primary radiation, theoretically, the average incident energy at the isocenter is shifted toward lower as the beam width is increased. Bujila et al compared ${\dot{K}}_{\text{air}}$ profiles along the z‐direction using a liquid ionization chamber and CT dose profiler (RTI Electronics, Mölndal, Sweden), and found an obvious difference in the two radiation profiles.^9^ One reason for this may be the energy dependence of the CT dose profiler.

Because the new technique can reduce scatter contamination and analyze the SPR, a few applications might be considered. At first, measurement of ${\dot{K}}_{\text{air}}$ is employed for determination of the HVL. McKenney et al. reported using the new technique to evaluate the HVL with a real‐time dosimeter and cylindrical aluminum cages with diameters of 122 and 505 mm.^6^ Systematic overestimation of the HVL was reported with the small aluminum cage because of scatter contamination in this geometry. Our beam width independent in‐air radiation output measurement may also be applicable to reduce the scatter in this geometry. Second, the ${\dot{K}}_{\text{air}}$ was recently used to assess the HVL in a dual‐source, dual‐energy CT system.^7^ Because the two tube potentials were concurrently used for this scanner, the probability of scattered radiation is complicated. Our beam width independent in‐air radiation output measurement enables scatter contamination to be analyzed and reduces the uncertainty of the ${\dot{K}}_{\text{air}}$ measurement. Third, Geleijins et al. provided the dose profiles along the z‐axis by Monte Carlo (MC) dose simulations for a 350‐mm‐long body phantom in axial scanning with a 160‐mm beam width.^2^ They then calculated the effects of both primary and scattered photons separately at different positions in the phantom. Since these data are derived by MC simulation, it is desirable to verify the data by measurement. However, to the best of our knowledge, no method has been described to measure both the primary and the scattered contributions separately. Our beam width independent in‐air radiation output measurement allows us to analyze the SPR and could be utilized for verification of the MC simulations. However, further studies are needed to systematically investigate the efficacy of the method.

The ${\dot{K}}_{0-\text{w}/\text{o}-\text{A}}$ and ${\dot{K}}_{0-\text{w}-\text{A}}$ are intended to be indices of radiation output for the purpose of QA in a wide‐beam CT scanner. These indices should be treated as fundamentally different dose quantities compared with the radiation dose to the patient, which depends on the clinically applied tube voltage, tube current, rotation time, beam width, and pitch in conjunction with the scanning length. However, Huda et al. reported conversion factors from ${\dot{K}}_{\text{air}}$ to effective dose or organ dose.^17^ At this point, the ${\dot{K}}_{0-\text{w}/\text{o}-\text{A}}$ and ${\dot{K}}_{0-\text{w}-\text{A}}$ might be utilized as well, although no definite claim can be made without verification. On the other hand, the unit of *K*_0_ is mGy/s, not mGy/rotation as for CTDI_vol_. The unit conversion is necessary to compare the ${\dot{K}}_{0-\text{w}/\text{o}-\text{A}}$ and CTDI_vol_; then, the measurement of gantry rotation time is worth considering.^18^

This study had some limitations. The length of the 6‐cc ionization chamber employed in the study was 38 mm. Therefore, beam widths less than 40 mm could not be measured with this ionization chamber. Bujila et al. used a 0.002‐cc liquid ionization chamber to characterize the dose profile along the z‐axis.^9^ Because the length of the chamber was 0.35 mm, the ${\dot{K}}_{0-\text{w}/\text{o}-\text{A}}$ and ${\dot{K}}_{0-\text{w}-\text{A}}$ for beam widths less than 40 mm could be measured.

De Denaro et al. reported the radiation dose profile free‐in‐air along the z‐axis measured by Gafchromic film and pointed out the obvious dose gradient (heel effect) along the z‐axis.^5^ Because the ${\dot{K}}_{0-\text{w}/\text{o}-\text{A}}$ and ${\dot{K}}_{0-\text{w}-\text{A}}$ indicate only the primary radiation output detected by the ionization chamber, the dose gradient along the z‐axis is not accommodated.

Finally, three different bow tie filters can be selected in the CT system. Because the ${\dot{K}}_{0-\text{w}/\text{o}-\text{A}}$ and ${\dot{K}}_{0-\text{w}-\text{A}}$ indicate the primary radiation output at the isocenter, these indices cannot characterize the peripheral radiation output. At this point, measurement of bow tie profiles using a real‐time dosimeter would be a pragmatic approach.^8^

## 5 CONCLUSION

A new beam width independent in‐air radiation output dosimetry in a modern CT system was introduced. Our new method can (a) measure the primary radiation output of the CT system, which is independent of the beam width; and (b) reduce scatter contamination, which affects the accuracy of ${\dot{K}}_{\text{air}}$ measurement. The new method requires neither the conventional 100‐mm‐long ionization chamber nor the CTDI PMMA phantoms, but only requires the farmer‐type ionization chamber. Our proposed dose index, ${\dot{K}}_{0-\text{w}/\text{o}-\text{A}}$, has the potential of providing information about the primary component of CT irradiations which cannot be acquired using other conventional dose metrics.

## CONFLICTS OF INTEREST

The authors have no relevant conflicts of interest to disclose.
